# Multifunctional mesoporous silica-cerium oxide nanozymes facilitate miR129 delivery for high-quality healing of radiation-induced skin injury

**DOI:** 10.1186/s12951-022-01620-5

**Published:** 2022-09-14

**Authors:** Daijun Zhou, Min Du, Han Luo, Fengwei Ran, Xiang Zhao, Yan Dong, Tao Zhang, Jie Hao, Dong Li, Jianjun Li

**Affiliations:** 1 Department of Oncology, General Hospital of Western Theater Command of PLA, Chengdu, 610083 China; 2 Department of Oncology, Southwest Cancer Center, Southwest Hospital, Army Medical University, 30 Gaotanyan Main St., Chongqing, 400038 China; 3 Department of Pulmonary and Critical Care Medicine, General Hospital of Western Theater Command of PLA, Chengdu, 610083 China

**Keywords:** Mesoporous silica, CeO_2_, miRNA-129, Radiation-induced skin injury

## Abstract

Radiation-induced skin injury (RISI) is an important challenge for clinical treatments. The main causes of RISI include hypoxia in the wound microenvironment, reactive oxygen species (ROS) activation, and downregulation of DNA repair proteins. Here, a multiple radioresistance strategy was designed for microRNA therapy and attenuating hypoxia. A novel mesoporous silica (MS) firmly anchored and dispersed cerium (IV) oxide (CeO_2_) nanoparticles to form MS-CeO_2_ nanocomposites, which exhibit superior activity in inhibiting radiation-induced ROS and HIF-1α activation and ultimately promote RISI wound healing. The miR129 serum concentrations in patients can promote radioresistance by directly targeting RAD17 and regulating the Chk2 pathway. Subsequently, MS-CeO_2_ nanocomposites with miR129 were conjugated with iRGD-grafted polyoxyethylene glycol (short for nano-miR129), which increased the stability and antibacterial character, efficiently delivered miR129 to wound blood capillaries, and exhibited low toxicity. Notably, nano-miR129 promoted radioresistance and enhanced anti-ROS therapeutic efficacy in a subcutaneous RISI mouse model. Overall, this MS-CeO_2_ nanozyme and miR129-based multiresistance radiotherapy protection strategy provided a promising therapeutic approach for RISI.

## Introduction

Radiotherapy is an effective method widely used in clinical tumor treatment. A major reason for the failure of radiotherapy is the damage to surrounding normal tissues by the applied radiation. For example, radiation therapy for head and neck malignancies often leads to radiation-induced salivary gland injury [1]. Radiation therapy to the chest for cancers of the lung, breast and esophagus predisposes to radiation-induced lung and heart disease [2]. Radiation therapy for pelvic or abdominal tumors often leads to radiation-induced gastrointestinal complications [3]. All these possible complications may not only lead to morbidity accompanied by a huge financial burden, but may also lead to suffering and significantly affect the quality of life of the patient [4]. It is necessary to develop new materials for reducing the radiosensitivity of normal tissues. 50% of cancer patients in North America and > 70% patients with malignant tumors receive radiotherapy, indicating radiation oncology’s critical role and status in cancer treatment [5]. Nevertheless, cutaneous radiation reactions are common adverse effects that limit radiotherapy dosage and treatment effects. In cancer patients receiving radiotherapy, up to 95% develop skin reactions and nearly 10% experience severe skin damage [6].

The exact mechanism of radiation-induced skin injury remains to be clarified and there is a lack of standardized and unified approaches for preventing and treating radiation-induced skin injury (RISI) [7]. There have been many biomaterial studies regarding RISI. Recently, Zhao [8] has suggested that fullerenol, known as a “free radical sponge,“ is an excellent choice for skin radiation protection due to its broad-spectrum free radical scavenging ability, good chemical stability, and biological safety. In vivo experiments have shown that medical sodium hyaluronate hydrogels containing fullerenol are suitable for dermal administration and can effectively protect epidermal stem cells and alleviate radiation dermatitis. Kyritsi [9] has designed a nonwoven patch composed of electrospun polymeric micro/nanofibers loaded with an aqueous extract of *Pinus halepensis* bark (PHBE), which has been prepared and clinically tested for its efficacy in preventing radiation dermatitis. Compared with control patches, PHBE patches exhibited significant anti-inflammatory activity and restored most skin parameters to normal levels one month after the end of radiation treatment. No adverse events were reported, suggesting that the application of PHBE patches could serve as a safe medical device for prophylactic radiation dermatitis treatment.

Cerium(IV) oxide nanoparticles (CeO_2_-NPs) have gained considerable attention in nanomedicine due to their promising applications in drug delivery, catalysis, biosensing, and medicine. These nanoparticles are relatively stable with special biocompatibility, little toxicity, low cost, and environmental friendliness [10]. Ce has two main oxidation states, including tetravalent (Ce^4+^) and trivalent (Ce^3+^) and thus ceria exists in the form of two oxides, namely Ce_2_O_3_ and CeO_2_, depending on the nature of the material. CeO_2_-NPs have a cubic fluorite structure with surfaces of intergrown Ce^3+^ and Ce^4+^. The main advantage of ceria (CeO_2_) is the creation of oxygen (O) vacancies in the crystal lattice [11]. Therefore, the redox properties of CeO_2_-NPs have been improved, contributing to the treatment of various oxidative stress-related diseases.

In the treatment of skin infections, nanosilica drug loading is a novel method for drug administration. Mesoporous silica (MS) is a promising drug carrier due to its specific surface area, large pore volume, high loading capacity and biocompatibility [12]. Traditional drug delivery systems produce several local adverse effects, such as burning sensation, skin irritation, greasy, stinging, pruritic rash, erythema and tenderness, thus making them unacceptable [4]. The proposed silica-NPs are ranked higher in their utility because they can overcome all the above problems associated with traditional treatment modalities. In the application of MS, there are no viscosity problems observed in conventional systems and such NPs have a relatively long action time and better penetration of drugs through the dermis without repeated use. Drug loaded mass spectrometry, when applied, can serve as a suitable carrier for drug delivery and an ideal for promoting wound healing [13]. In particular, silica increases re-epithelialization rates by enhancing cell replenishment, neovascularization and epidermal maturation, thereby accelerating wound closure and contraction [14].

MicroRNAs (miRNAs) are short non-protein-coding RNAs composed of 18–25 nucleotides. Progress has been made in understanding the role of miRNAs in epigenetic regulation, in which miRNAs regulate protein concentrations of target mRNAs. A previous study has found that miR129-5p is downregulated in gastric cancer and inhibits cell proliferation by negatively regulating HMGB1 [15]. Qiu et al. [16] have found that HS-MC-Exo enriched with miR129 significantly reduces the inflammatory response and chondrocyte apoptosis, whereas HS-MC-Exo depleted of miR129 increases IL-1β-Mediated inflammatory response and chondrocyte apoptosis. In terms of mechanism, miR129 targets the 3’UTR end of HMGB1 and inhibits IL-1β-mediated upregulation of HMGB1.

However, the relationship between wound healing and miR129 in the progression of RISI has not been elucidated. Silicon (Si)-based sol-gel NPs do not support bacterial growth in the wound bed and exhibit good tissue response without causing inflammation. Therefore, the current study was aimed to investigate the MS for loading a combination therapy to promote rapid and efficient wound healing and present these dual drug-loaded silica-NPs as an alternative option to the routinely available strategies [17].

In this study, a sprayable MS-CeO_2_-miR129 nanozyme was successfully fabricated. The ability of these nanozymes for promoting proliferation, migration, angiogenesis and anti-apoptosis of HaCaT and HUVEC cells was confirmed, thus endowing prepared nanozymes with synergistically anti-radiation ability in vitro and in vivo. Thoralf Bernhardt et al. [18] describes a radiation shielding.

chamber was successfully constructed allowing selective irradiation of the right hind leg. A moderate radiodermatitis is induced with irradiation doses in the range of 60–70 Gy under the here described conditions. Symptoms peak about 8 days after irradiation and decrease relatively quickly thereafter. Histological analyses confirmed typical signs of inflammation. Ji-Hee Kim et al. [19] investigated whether 2-Methoxyestradiol can repair radiation-induced vascular damage.Macroscopic vascular damage occurred 5 days after 17 Gy dorsal skin irradiation. H&E and Masson’s Trichrome staining showed that irradiation increased skin inflammation, skin thickness, and collagen deposition, but these were significantly recovered by post-treatment with 2-Methoxyestradiol after irradiation. So,the mutual promotion relationship between MS, CeO_2_ and miR129 was also examined and a RISI mouse model established to examine the effects of MS-CeO_2_-miR129 nanozymes on wound healing. In addition, the related mechanism of nanozyme bioactivity was closely analyzed. This interesting study was believed to offer an important reference for the biomaterial therapy of RISI.

## Materials and methods

### Ethics statement

HaCat and VEGF cells were provided by the Department of Oncology, Southwest Hospital. The Experimental Animal Department of the Army Medical University provided male BALB/c mice of ~ 25 g in weight that were individually fed in plastic cages in a 12 h circadian rhythm with a relative humidity of 50% and an ambient temperature of 25 °C under typical circumstances. Animal experiments were approved by the animal ethics committee of General Hospital of Western Theater Command of PLA, and all operations were performed following relevant ethical regulations.

### Preparation of MS-CeO_2_-miR129

SiO_2_ and Cerium nitrate was prepared by RuiXi Materials Tech Co. (Xian, China). Anti-IFI6, Anti-HIF-1α was purchased from Bioss Co. (Beijing, China). Anti-SSBP1 was purchased from FineTest Co. (Wuhan, China). miR129 was purchased from GenePharma Co. (Shanghai, China), sense strand: CUUUUUGCGGUCUGGGCUUGC, antisense strand: AAGCCCAGACCGCAAAAAGUU. The end of miR129 is modified by NH2 group and has green fluorescence.

### Synthesis of MS

N,N,N-Trimethylhexadecan-1-aminium 4-methylbenzenesulfonate(CTATos), Triethanolamine (TEAH3), H_2_O, added to a 50 ml flask, mixed, and then heated to 80℃ with stirring until completely dissolved. Add tetraethyl orthosilicate (TEOS), stop the reaction at 80℃ for 2 h and cool, (12,000 rpm, 10 min) collect by centrifugation, wash 3 times alternately with ddH_2_O and ethanol and redisperse in ethanol for further use. The obtained nanoparticles were dispersed in ethanol and then added to the mixed solution of ethanol/hydrochloric acid = 10/1 for overnight reaction at 70 °C. It was collected by centrifugation (12,000 rpm, 10 min), and H_2_O and ethanol were alternatively washed 3 times, and finally toluene washed 2 times and water washed 3 times, redispersed in water, and used for further use.

### Preparation of CeO_2_

3.12 ml TEOS was added quickly and reacted at 80 ℃ for 2 h. Cerium nitrate was added to the mixed solution of water/ethylene glycol, after heating to 60℃, ammonia water was added, and after reaction for 3 h, it was washed by centrifugation with water and redispersed in water for further use.

### Preparation of DSPE-PEG-miR129

Dissolve 1 mg of DSPE-PEG-NHS in 1ml sterile PBS(pH = 8), add 20 OD miR129-NH2 and incubate overnight at room temperature; then dialyze DSPE-PEG-miR129 purify and remove unreacted DSPE-PEG-NHS and miR129-NH2.

### Preparation of MS-CeO_2_-miR129

DSPE-PEG-miR129 was dissolved in 1 ml water, 900 UL was added to the SiO_2_ dispersion and after stirring overnight, CeO_2_ was added to stir overnight. Then, the pellets were washed by centrifugation, dispersed in water and stored in 4 ℃ protected from light. To determine the encapsulation efficiency (EE) and loading efficiency (LE) of miR129, different w/w ratios of MS-CeO_2_ and SPE-PEG-miR129 were combined. The supernatant of the last step was centrifuged and mixed with 5× loading buffer and agarose gel electrophoresis was performed in 1% agarose gel with Gelred-containing TBE buffer at 80 V for 45 min. The miRNA bands were analyzed using BioRad imaging system. The EE and LE of miR129 in MS-CeO2-miR129 were calculated by the following equations:

$${\text{LE}} (\%)={\frac{\text{weight of miR129 in MS-CeO2-miR129}}{{\text{weight of MS-CeO2}}}}.$$

$${\text{EE}} (\%)={\frac{\text{weight of miR129 in MS-CeO2-miR129}}{{\text{weight of miR129}}}}.$$

### Characterization

Transmission electron microscopy was used to observe the morphology of the MS-CeO_2_-miR129. The particle size and zeta potential were evaluated using dynamic light scattering. Fourier transform infrared (FTIR) spectra were recorded using a Nicolet 6700 FTIR spectrometer (4000–600 cm^− 1^). UV-Vis-NIR measures the UV spectrometer/near-infrared light absorption effect of materials. Energy Dispersive Spectroscopy(EDS)/X-ray Photoelectron Spectroscopy(XPS) analyzed the elemental composition of the material. In vitro H_2_O_2_ test is used to verify the ability of the material to decompose H_2_O_2_ per unit time.

### Biocompatibility evaluation and cell morphology observation

Cytological experiment was divided into 5 groups, Group A was normal cell group, Group B was normal cell + 3 Gy radiation group, Group C was normal cell + 3 Gy radiation + MS group, Group D was normal cell + 3 Gy radiation group + MS-CeO_2_ group and Group E were normal cells + 3 Gy radiation + MS-CeO_2_-miR129 group. Cytotoxicity was evaluated using CCK-8 assays. HaCat cells were seeded onto Groups A–E at a density of 2 × 10^3^ cells/well in DMEM supplemented with 5% fetal bovine serum and 1% penicillin/streptomycin at 37 ℃.

After incubation for 0, 3 and 6 days, cell viability was quantified using CCK-8 assays .To study the influence of cytoskeleton morphology, Human vascular endothelial cells (HUVEC) were seeded on the medium of group A-E for 24 h. Cells were then washed 3 times with pre warmed phosphate buffered saline and fixed with pre warmed 4% paraformaldehyde at room temperature. We rinsed fixed cells in phosphate buffered saline and soaked them. Cells were then incubated with as prepared phalloidin for 30 min at 37 °C without light and with DAPI (2-(4-amidinophenyl)-6-indolecarbamidine dihydrochloride) for 5 min. Fluorescence images of stained cells were obtained using a confocal laser scanning microscope (780, Zeiss, Germany). Then, HUVECs were stained with DAPI and phalloidin, followed by fluorescence observation.

### Scratch wound migration

HaCaT were grown to confluence in complete medium and seeded in 24 well plates (2 × 10^4^/well). Across the monolayer (0 h) with a pipette, tip and a Zeiss video microscope was employed for 24-h inspection. For a single experiment with five replicates per Group, ImageJ 1.48v software (NIH, USA) was used for specific measurements. These assays were performed in triplicate.

### In vitro tube formation assay

Briefly, mixing 2 × 10^4^ HaCaT cells were seeded onto Matrigel coated 96 well plates and then added to Groups A-E. After 24 h of stimulation, newly formed tubes were photographed with an inverted phase microscope (Olympus, USA). There were five replicates per Group. Each experiment was performed three times.

### Flow cytometry

Cell apoptosis assays were performed using a flow cytometry assay and the Annexin V-FITC/PI Apoptosis Detection kit (Dojindo Molecular Technologies, Japan). Briefly, cells were collected and suspended in 200 µl of binding buffer containing 5 µl of Annexin V-FITC and 5ul of PI.The cells were analyzed using Cell Quest software using Fluorescence Activating Cell Sorter (FACS) (Beckman Coulter, USA).

### Cell clone formation assay

Methods were consistent with 2.8. Each well was stained with 1ml 0.005% crystal violet for 1 h, and the number of communities was calculated by Image Pro Plus 6.0 after imaging.

### Bacterial co-culture

MRSA(methicillin resistant *Staphylococcus aureus*) and ***E. coli*** (*Escherichia coli*) were obtained from the Army Medical University. The bacteria were amplified (grown overnight) to 1 × 10^9^ colony-forming units (CFU)/mL and then diluted with Luria-Bertani medium to 1 × 10^4^ CFU/mL. Draw the same amount of bacterial liquid to coat the plate. After incubation at 37 °C for 24 h, the change in bacteria number was determined.

### The RISI mouse model

Each mouse was anesthetized by intraperitoneal injection with approximately 0.2 ml pentobarbital. The linear accelerator emits 6 Mev electron rays (once irradiation, 30 Gy, irradiation field 1 cm × 1 cm, dose 300 cGy/min, 10 min). The source skin distance was 1 m, and the remaining skin was blocked using a lead plate. After each group was irradiated, the irradiated part was covered with material and replaced every other day for a total of 7 to 14 days after irradiation and divided into five groups, five mice in each group. Cytological experiment was divided into 5 groups, Group A was normal mouse group, Group B was normal mouse + 30 Gy radiation group, Group C was normal mouse + 30 Gy radiation + MS injection group, Group D was normal mouse + 30 Gy radiation + MS-CeO_2_ injection group and Group E were normal mouse + 30 Gy radiation + MS-CeO_2_-miR129 injection group. The wound areas before and after healing were compared to calculate the healing rate, and IPP6.0 software was employed. The wound-healing rate = (the initial wound area – the wound area after healing for a specific time)/the initial wound area × 100%. The RISI rating followed the Douglas and Fowler scoring method.

### Hematoxylin–Eosin (HE) staining and histological analysis

Wound samples from each mouse were taken at 14 days after wounding to produce paraffin sections for HE staining. High quality images were selected for HE staining. Several pathology experts measured the length of the Neo-epithelium in a blinded fashion.

### Western Blot and immunohistochemical staining detection

Fourteen days after the injury, small squares of 10 mm × 10 mm were sampled from the full-thickness defects, including both new epidermis and granulation tissues, and were then frozen immediately in liquid nitrogen. A bicinchoninic acid approach was used to determine the protein concentration of supernatants, referring strictly to the manufacturer’s instructions (Varioskan Flash; Thermal Scientific, USA). Antibodies were diluted at 1:1000. Goat anti-rabbit secondary antibody labeled with horseradish peroxidase (Zhongshan Biology Company, China) was diluted to 1:2000. PVDF(polyvinylidene fluoride) membranes were harvested and sent for chemiluminescence observation (Thermal Scientific, USA).In terms of immunohistochemistry, Wound tissue sections were deparaffinized and rehydrated, and heat mediated antigens were retrieved by incubation at 95 °C in sodium citrate buffer. Finally, we obtained images using a light microscope (Leica, Germany, ctr6000).

### Statistical analysis

Each experiment was performed in triplicate, and all data are presented as mean ± standard error of the mean. Two groups were compared using the Student’s t-test, and differences were considered statistically significant at P < 0.05.

## Results and discussion

### Fabrication of MS-CeO_2_-miR129

**Fig. 1:**
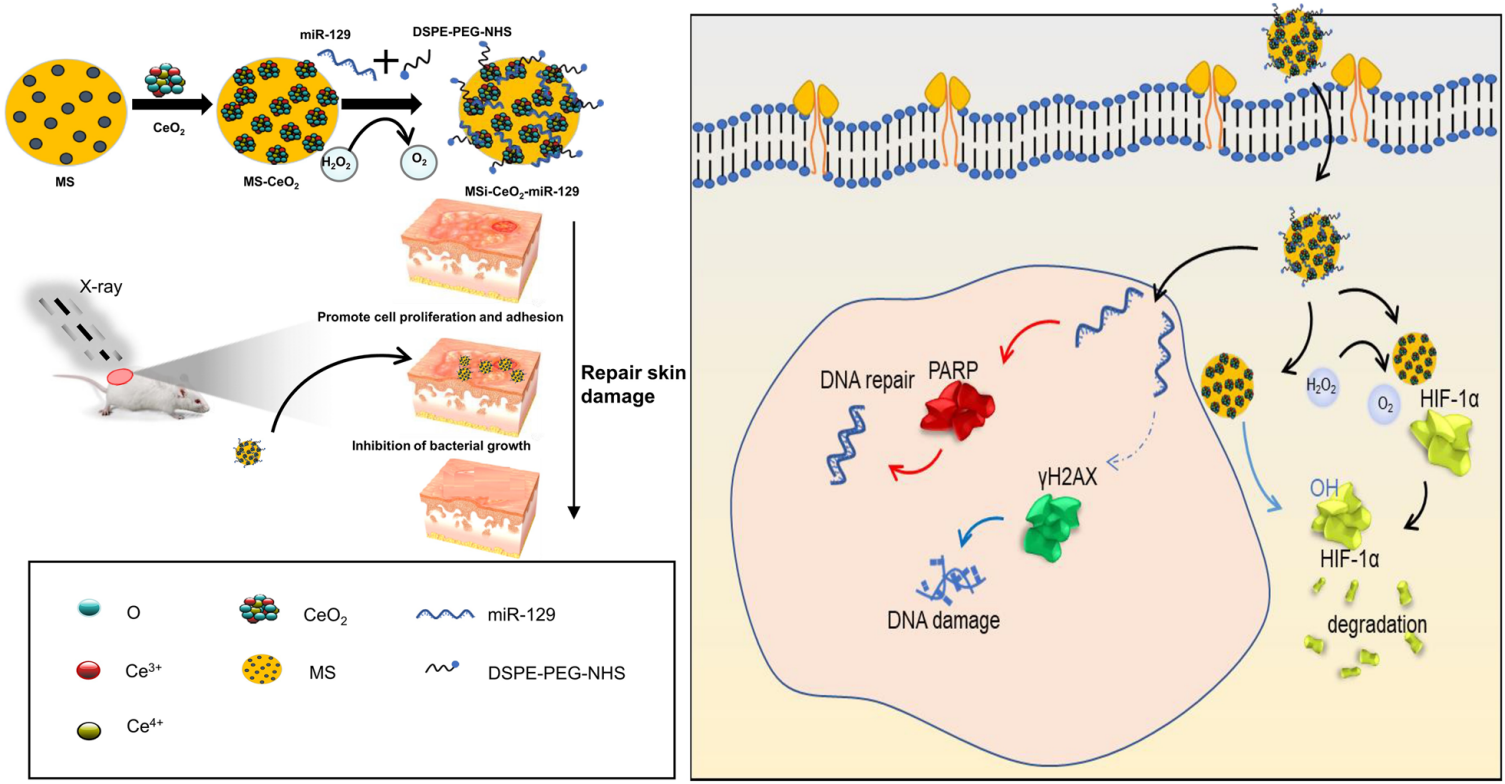
Schematic illustration showing the fabrication of MS-CeO_2_-miR129 and its application for RISI healing

To meet the needs of RISI wounds and the complex process of wound healing, a multifunctional composite nanomaterial was prepared which possessed excellent reduced radiosensitivity, moisture retention and biological safety along with anti-ROS and anti-hypoxia capabilities. The fabrication process of MS-CeO_2_-miR129 is shown in Fig. 1.

CeO_2_-NP nanozymes were immobilized on MS surfaces to obtain good catalase activity and prevent their aggregation. MS-CeO_2_ exhibited remarkable anti-ROS and HIF-1α abilities at physiological pH or weak acidic wound microenvironments. HIF-1α can significantly promote wound healing and reduce the curative effect of skin radiotherapy. Moreover, both MS and miR129 are negatively charged, such that the presence of positively-charged CeO_2_ NPs decreased their electrostatic repulsions. The miR129 was loaded by MS-CeO_2_ because miR129 can reduce DNA damage-induced apoptosis by directly targeting RAD17 and regulating the CHK2 pathway. The above composite, MS-CeO_2_-miR129-polyethylene furonate (PEF)-iRGD (peptide), referred to here as nano-miR129, was then encapsulated with polyethylene glycol (PEG)-iRGD to improve its dispersity and stability and to protect miRNAs from RNases. Taken together, nano-miR129 has the advantages of nanozymes and miRNA therapeutics.

### Morphology and composition characterization of MS-CeO_2_-miR129

**Fig. 2:**
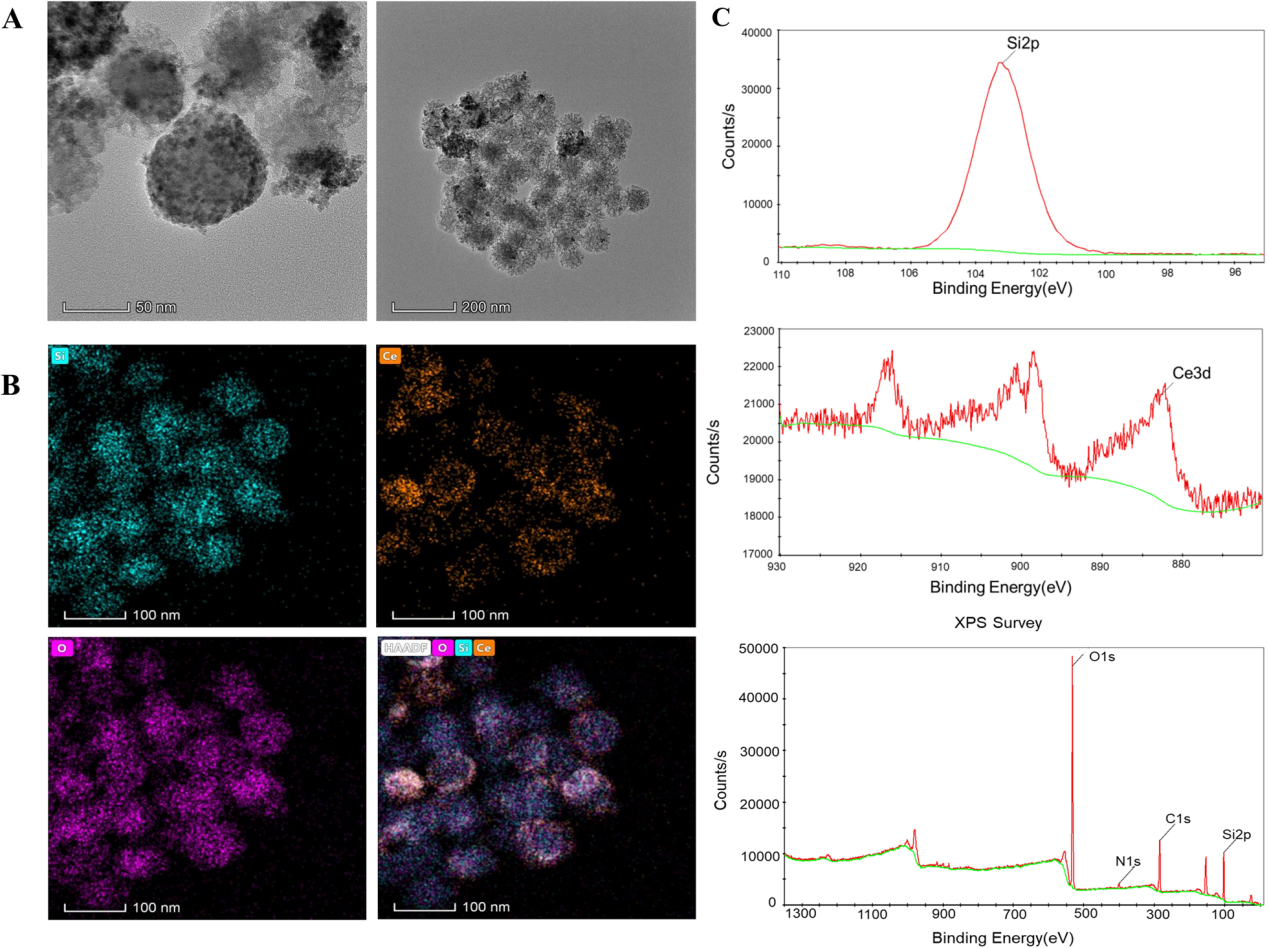
Characterizations of MS-CeO_2_-miR129. SEM of MS-CeO_2_-miR129 (**A**). EDS images of MS-CeO_2_-miR129 (**B**), including Si, Ce and O. XPS of MS-CeO_2_-miR129 (**C**). Test elements included O, nitrogen, Si and carbon

Characterization by TEM imaging of CeO_2_-miR129-NP after encapsulation with the MS shell is shown in Fig. 2. Characterization of the MS-CeO_2_-miR129 nanocomposites indicated that the final composite size was 60–100 nm (Fig. 2a). SEM image analysis revealed that none of the CeO_2_-miR129-NPs were outside the shell and comparison of the elemental distribution from energy dispersive X-ray analysis (EDS, Fig. 2B) confirmed the crystalline structure of CeO_2_-miR129 cores inside the shell. These results indicated that ceria had been successfully immobilized into MS.

Robert [20] has reported that the ultraviolet-visible (UV-Vis) spectra of CeO_2_-NPs core before and after mSiO_2_ coating are similar, confirming that the initial CeO2-NPs coating was successful and NP aggregation or degradation not induced. Importantly, in contrast to uncoated CeO_2_-NPs, mSiO_2_ coating allowed colloidal stability in physiological media. Zuo [21] have suggested that EDC chemistry can be used to connect carboxyl-functionalized CeO_2_-NPs with drug-loaded amine-functionalized MS. TEM and SEM images showed that MS surfaces were clean and smooth. Although discrete black spots on MS surfaces can be clearly seen in the presence of CeO_2_, high-resolution TEM also demonstrated that drug-loaded MS successfully tethered 4 nm CeO_2_-NPs to the surface of the drug carrier.

In addition, the Ce valence states on MS-CeO_2_-miR129 surfaces were explored by X-ray photoelectron spectroscopic (XPS) analysis (Fig. 2C). The resulting XPS peaks at 885.1 and 903.2 eV in MS-CeO_2_-miR129 were assigned to Ce^3+^ and peaks at 881.7, 888.1, 898.1, 900.9, 906.4 and 916.4 eV assigned to Ce^4+^ in MS-CeO_2_-miR129. These results contra-indicated the coexistence of mixed valence Ce in MS-CeO_2_-miR129, which can act as an ROS scavenger due to oxidation of Ce^3+^ to Ce^4+^. Zuo [21] has also reported that XPS results shows the typical absorption peaks of CeO_2_. XPS spectroscopic analysis clearly here showed the presence of CE-3*d* and Si-2*p* and, more importantly, XPS spectra in the Ce region showed redox activity due to the simultaneous presence of Ce^3+^ and Ce^4+^ oxidation states at binding energies from 880 to 920 eV.

### Physical and chemical characterization of MS-CeO_2_-miR129

**Fig. 3:**
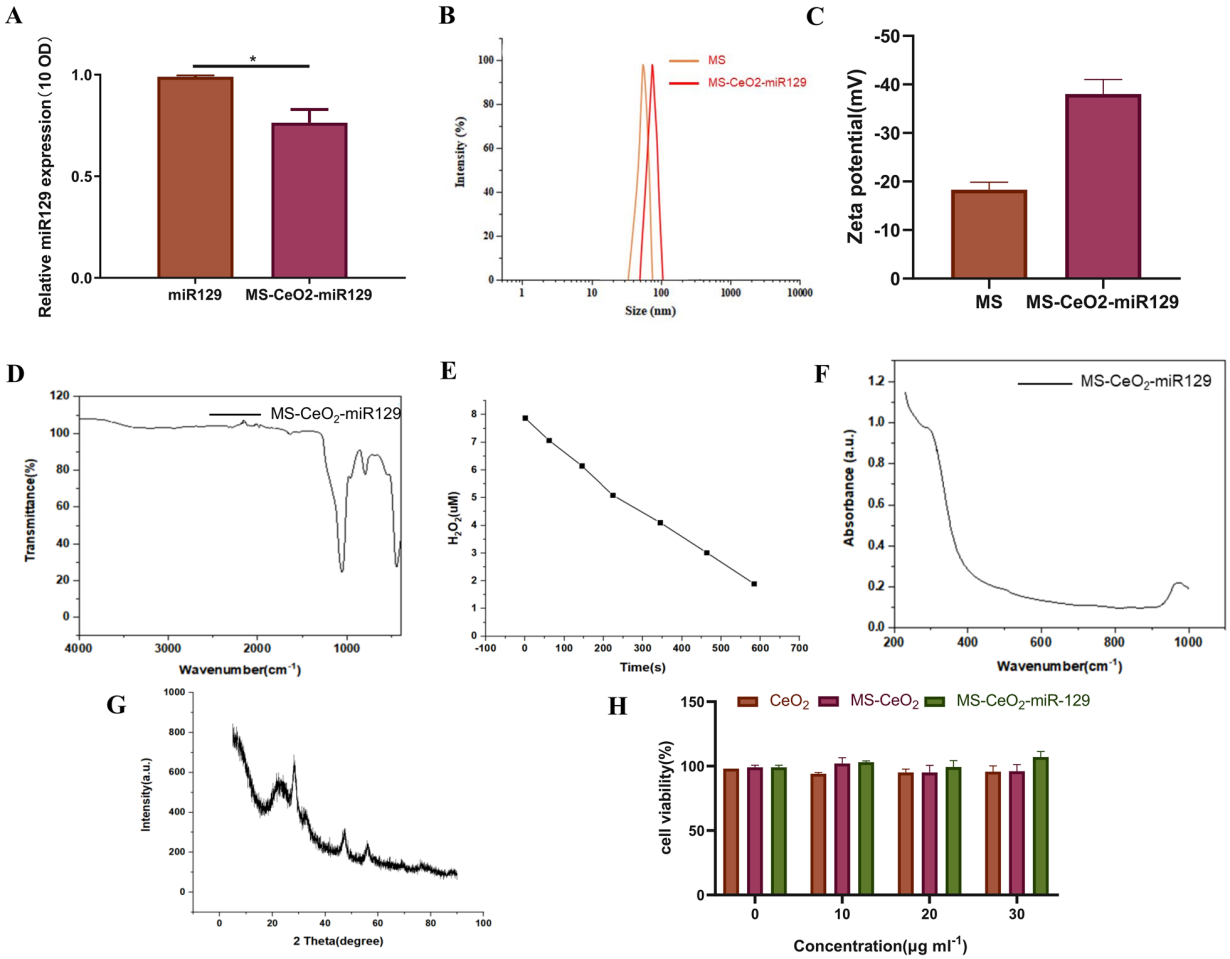
Characterizations of MS-CeO_2_-miR129. Relative miR129 expression of miR129 and MS-CeO_2_- miR129 (**A**). Particle size and potential analysis (**B**), with size at 229 n, PDI at 0.119, and zeta potential at − 38.60 mV (**C**). FT-IR of MS-CeO_2_-miR129 (**D**). Catalytic capacity of MS-CeO_2_-miR129 for H_2_O_2_ in vitro (E). UV spectrum of MS-CeO_2_-miR129 (**F**). XRD of MS-CeO_2_-miR129 (**G**). The cytotoxicity of nanoparticles with the indicated modifications was evaluated by Cell Counting Kit-8. The data are presented as the mean ± SD (**H**)

The loading of miR129 on MS-CeO_2_ was further verified using TaqMan qPCR analysis of miR129 expression in miR129 and MS-CeO_2_-miR129 at the same optical density (OD). The results showed that miR129 was successfully loaded on MS-CeO_2_ and the loading efficiency reached ~ 77% (Fig. 3A). Due to the difference in surface area-volume ratio, the adsorption capacity is also related to the nanoparticle size, and the amount of adsorbed RNA decreases as the mesoporous silica (MPS) nanoparticle size increases [1]. We optimized and determined the reaction concentration of 1 mg of DSPE-PEG-NHS dissolved in 1 ml of sterile PBS (pH = 8) with the addition of 20 OD miR129-NH2. Under the optimized reaction conditions, particle size and zeta potential analyses indicated the particle size is 85 nm, polydispersity index (PDI) is 0.119, and zeta potential is -38.60 mV (Fig. 3B, C) .

Drug loading and capping processes were also probed through Fourier transform-infrared (FT-IR) and UV-Vis-near-IR (NIR) spectroscopies, which showed that the typical absorption peaks of CeO_2_ and MS were displayed in MS-CeO_2_-miR129 particles (Fig. 3D, F).

MS-CeO_2_-miR129 was able to decompose 85% of aqueous hydrogen peroxide (H_2_O_2_)in 10 min and exhibited strong catalytic ability to H_2_O_2_ (Fig. 3E). Zuo [21] has exploited the redox-susceptible nature of CeO_2_ gatekeepers. Drug-loaded and CeO_2_-encapsulated monodisperse silica microspheres were exposed to different concentrations of H_2_O_2_ and a controllable and responsive dual drug release observed. The monodisperse silica microspheres released 63 and 72% of the drug in 6 h under the action of 10 and 20 mM H_2_O_2_ buffers, respectively, while the hydrophobic dye IR780 was slowly released under this action. The H_2_O_2_ concentration clearly affected release of the drug, with drug release in 20 mM H_2_O_2_ higher than in 10 mM H_2_O_2_, which is related to the etching speed of CeO_2_-NPs. Robert [20] has evaluated whether an MS coating affected the antioxidant capacity of CeO_2_-NPs. The study found that cell viability increased from 86 ± 1.1% (H_2_O_2_-stimulated cells) to 91 ± 1.1% (CeO_2_-NPs) and 94 ± 2.6% (CeO_2_@mSiO_2_). None of these effects were observed when pure mSiO_2_-NPs were used. When free CeO_2_NPs were used, ROS levels were reduced by 60%. CeO_2_@mSiO_2_ antioxidant activity was significantly improved by two-fold and resulted in complete recovery of the basic ROS value. High angle X-ray diffraction (XRD) patterns of MS-CeO_2_-miR129 and related nanoformulations matched well with the cubic character of standard CeO_2_ (Fig. 3G). The observed broadening of XRD peaks was related to decreased CeO2-NP particle size. Zuo [21] has suggested that the BET specific surface area of samples before and after CeO_2_ coverage, calculated by nitrogen adsorption-desorption, and found that the BET specific surface area of the samples decreased sharply from 893.8 (MS) to 228.3 cm^2^/g (CeO_2_@MS). The cytotoxicity of nanoparticles is particularly critical for medical applications. To test the cytotoxicity of MS-CeO2-miR129, we measured the viability of HaCaT cells cultured with different concentrations of CeO2, MS-CeO2 and MS-CeO2-miR129. We observed no significant toxicity of MS-CeO2-miR129 in HaCaT cells (Fig. 3H).

### Biocompatibility and antibacterial activity of MS-CeO_2_-miR129

**Fig. 4:**
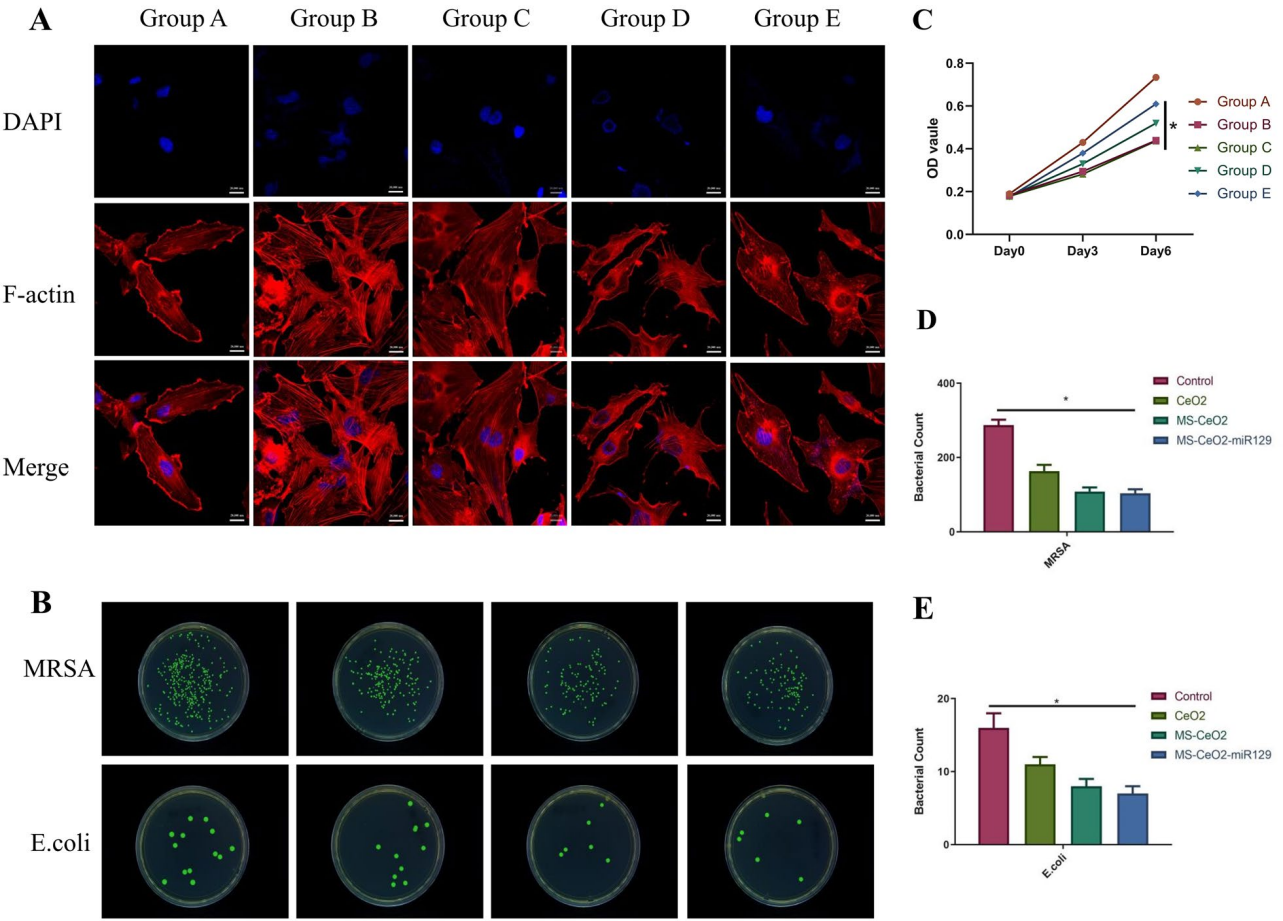
Biocompatibility and antibacterial activity of MS-CeO_2_-miR129. FITC/DAPI staining images of HUVEC cells cultured on MS-CeO_2_-miR129 on day 7 (**A**). Antimicrobial activity of G+(MRSA) and G-(E.coli) bacteria (**B**). CCK-8 assay of HaCaT cells cultured on MS-CeO_2_-miR129 on day 7 (**C**). Bacterial count in **B** (**D**, **E**). * *p* < 0.05

HUVEC cells and MS-CeO_2_-miR129 were co-cultured for 7 d and subsequent FITC/DAPI staining showed that cell morphology was not significantly different from normal Group, with the nuclei and cytoplasm intact (Fig. 4A). The CCK-8 method showed that, on the 3th day, the ODs of HaCaT cells in Groups B and E were reduced after 3 Gy of irradiation, suggesting that radiation significantly inhibited cell growth (Fig. 4C). On the 6th day, the OD of Group E recovered significantly and was significantly higher than that of Group B *(p <* 0.05), suggesting that MS-CeO_2_-miR129 might not affect the growth of irradiated cells in the short term (1–3 d), while the growth curve of irradiated cells returned to normal in the long term (> 6 d). Furthermore, the obtained data were consistent with previous data that cell viability loss is dose/concentration dependent.

In previous studies [22], CeO_2_-NPs have been reported to be toxic to cancer cells but did not show any toxicity toward normal cells. Moreover, CeO_2_-NPs at high concentrations have been found to have no toxic effect on normal cells. A previous study [23] has also reported the use of 400 µg/ml CeO_2_ to evaluate anticancer effects on human lung cancer lines. These findings suggest that CeO_2_-NPs toxicity is only specific for cancer cells and they might be safer for in vivo studies, in medicine and industry. At a low of CeO_2_-NP concentration (25 µg/ml), cells begin to shrink and, similarly, with increasing concentration (75 µg/ml), there are further changes in cell morphology suggestive of apoptosis. At a concentration of 100 µg/ml, cancer cells exhibit increased apoptosis[10].

Methicillin-resistant *Staphylococcus aureus* (MRSA, gram positive, G^+^) and *Escherichia coli* (G^−^) in the control group grew well, but CeO_2_ effectively inhibited bacterial growth (Fig. 4B, D, E). MS-CeO_2_ better inhibited bacterial growth, but MS-CeO_2_-miR129 did not show better inhibition or bacteriostasis. This might have been related to the fact that miR129 has not been reported to exhibit antibacterial activity, but CeO_2_ has been reported to have good antibacterial activity. A study by Ahmed [10] study has shown that the antibacterial efficacy of CeO_2_-NPs against sea anemones largely depends on the NP concentration. As the concentration increases, the inhibition range also increases. In an antibacterial study, *S. aureus* (G^+^) has been observed to be relatively more sensitive to CeO_2_-NPs than *Klebsiella pneumoniae* (G^−^). This suggests that G^+^ bacteria are more sensitive to ROS, which might be the reason why *S. aureus* strains have a wider inhibition range.

Ma [24] has suggested that, by combining the inherent antibacterial activity of CeO_2_-NPs, MoS_2_-CeO_2_ nanocomposites exhibit excellent antibacterial ability for photothermal therapy under 808 nm laser irradiation against both G^+^ and G^**−**^ bacteria, thereby reducing the risk of wound infection. In vitro antibacterial tests have shown that a 5-mol% CeO_2_ bioactive glass/gelma hydrogel composite exhibited good antibacterial properties [11].

### In vitro cytological study of MS-CeO_2_-miR129

**Fig. 5:**
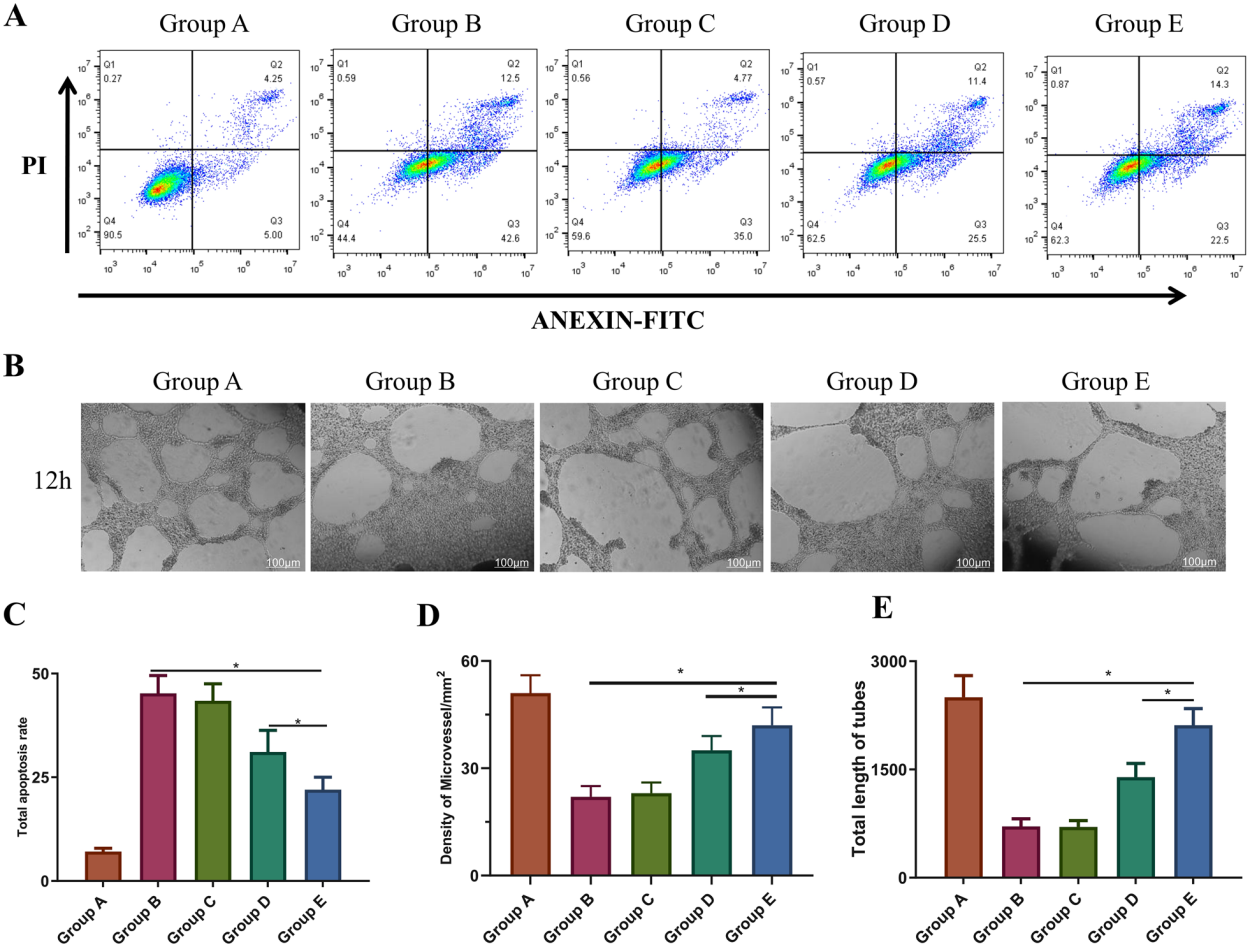
In vitro cytological study of MS-CeO_2_-miR129. Flow cytometry and total apoptotic rate results (**A**, **C**). In vitro tube formation assay (**B**). Number of nodes and total length of tubes in **B** (**D**, **E**). * p < 0.05

Flow cytometry assays were performed to further examine the potential effects of MS-CeO_2_-miR129 on apoptosis. Radiation significantly increased the apoptotic rate (Fig. 5A, C). Compared with Group B, Group E had a lower apoptotic rate *(p <* 0.05) and the effect on Group E was higher than on Group D. This suggested that MS-CeO_2_-miR129 significantly reduced the apoptotic rate and that increased miR129 in Group E played a vital role in regulating apoptosis. These effects might have been related to anti-ROS mechanisms. CeO_2_-NPs have good ROS scavenging ability due to the existence of the reversible conversion of Ce^3+^ and Ce^4+^ on the surface of CeO_2_-NPs.

In Ma’s experiments [24], the supporting information shows that ROS in cells can be effectively eliminated when co-cultured with CeO_2_-NPs. The adsorption of PEG-MoS_2_ nanosheets onto CeO_2_-NPs gives the MoS_2_-CeO_2_ system good ROS-scavenging ability, among which MoS2-CeO_2_ nanocomposite performs the best. In this study, MS was used as the benchmark for the MS-CeO_2_ concentration, such that MS-CeO_2_ can load much more CeO_2_-NPs than it can CeO_2_, giving MS-CeO_2_ nanocomposites the best antiapoptotic ability. The good anti-apoptotic ability of the MS-CeO_2_ system indicated that this material attenuated the inflammatory response and thus would promote healing around wounds and especially around RISI wounds. Previous studies have reported that miR129 has the potential to prevent renal fibrosis and tubular epithelial mesenchymal transition, suggesting that miR129 might provide a promising therapeutic strategy for renal injury [25]. In addition, one study has shown that KCNQ1OT1 and JAG1 are upregulated in non-small cell lung cancer (NSCLC) tissues and cells, while miR-129-5p is downregulated. Knockdown of KCNQ1OT1 significantly inhibits NSCLC cell proliferation, migration and invasion. Meanwhile, miR-129-5p inhibition reverses the effects of KCNQ1OT1 downregulation on NSCLC progression [26].

In the process of RISI wound healing, new capillaries grow toward the wound at an astonishing rate and produce a large neovascular network with a density 2, 3, and even 10-fold that of normal tissue. Endothelial cells are essential cells of blood vessels and play an important role in the vascularization process. HUVEC migration and tube formation assays have been performed to evaluate the angiogenic activity of some materials [11]. The effects of MS-CeO_2_-miR129 on tube formation of HUVECs is shown in Fig. 5B, D, E. Matrigel ascending angiogenesis experiments were carried out for ~ 24 h after co-cultivating HUVECs with materials of different groups. The number of blood vessels of normal cells in Group A was the largest and the length of the blood vessels the longest. The formation of blood vessels was significantly restricted after 3 Gy of irradiation in Group B, while the MS-CeO_2_ precursor material in Group D partially restored blood vessel formation *(p <* 0.05). The best pro-angiogenesis effect was in Group E (*p <* 0.05). The composite MS-CeO_2_-miR129 promoted tube formation by HUVECs, which was a challenge in RISI trauma induced by hypoxia and high oxidative stress. Further data from Chen’s [11] study has shown that CD31 and α-SMA immunofluorescence staining is clearly enhanced. Therefore, the present study successfully demonstrated that MS-CeO_2_-miR129 indeed promoted angiogenesis.

### In vitro cytological study of MS-CeO_2_-miR129

**Fig. 6:**
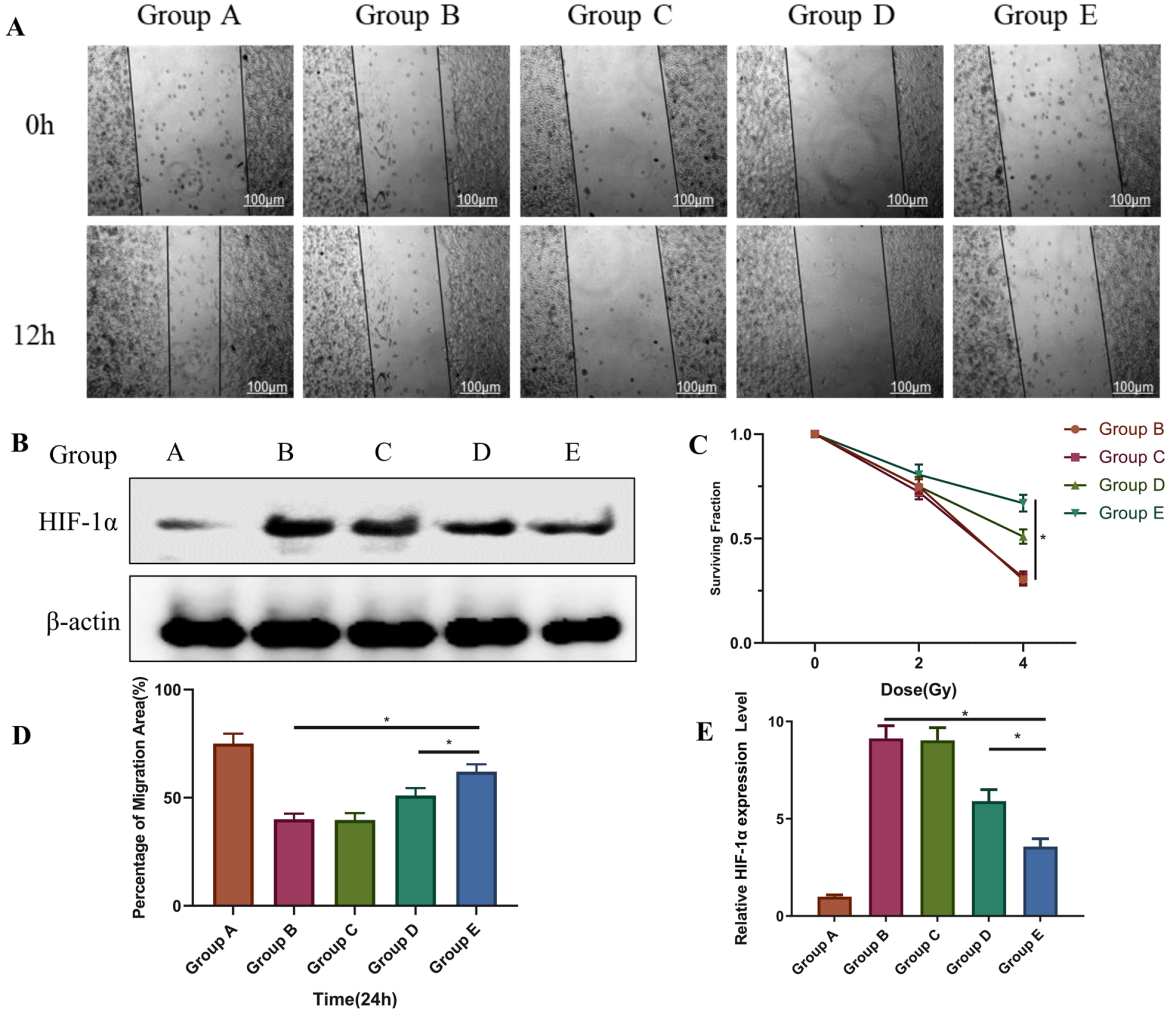
In vitro cytological study of MS-CeO_2_-miR129. Scratch wound migration of HaCaT cells with MS-CeO_2_-miR129 (**A**). Western Blot of HIF-1α and semi-quantitative analysis (**B**, **E**). Cell clone formation assay (**C**). Percentage of migration area in **C** (**D**). * *p* < 0.05

If a material can promote cell migration and proliferation in the proliferation phase of wound healing, it will be very beneficial for promoting wound healing. Here, HaCaT cells were co-cultured with materials in the various groups for ~ 24 h and then subjected to cell scratch tests (Fig. 6A, D). The 24-h migration rate of Group B was significantly reduced (*p <* 0.05), which was possibly related to radiation effects on cell migration, but migration inhibition of Group E decreased significantly, suggesting that the material increased the migration rate of irradiated cells *(p <* 0.05). Notably, the cell migration rate of Group E was higher than that of Group D (*p <* 0.05), suggesting that increased miR129 expression promoted the migration of HaCaT cells. Compared with the control group, both MS-CeO_2_ NPs and MS-CeO_2_-miR129 nanocomposites showed some promotion on cell migration. As MS-CeO_2_-miR129 possessed the best biocompatibility and better antioxidant properties, its effect of promoting cell migration was the best compared with the other groups. For a more intuitive demonstration, the healing rate of the scratched area was quantitatively calculated using image J software. Ma [24] has shown that cell scratch coverage can reach 89% after 48 h with the addition of MoS_2_-CeO_2_ nanocomposites in the culture medium, which is much higher than that of CeO_2_-NPs (59%) and PEG-MoS_2_ nanosheets (54%). The present cell scratch assays showed that the combination of MS-CeO_2_ nanosheets with miR129-NPs effectively promoted cell migration, which is essential for wound healing.

In RISI, hypoxia causes adenosine triphosphate (ATP) depletion and, once the O_2_ supply is restored, Increased ATP produced by mitochondria, inducing apoptosis. If a hypoxic environment is continuously maintained, cell necrosis occurs, which causes RISI damage. HIF-1α expression in Western blotting analysis showed that expression of HIF-1α in Group E was significantly lower than that of Groups B (p < 0.05) and D (p < 0.05), suggesting that the material can reduce HaCaT cell hypoxia by inhibiting the HIF-1α pathway (Fig. 6B, E). Notably, it has been reported that knockdown of MEG3 can enhance the cerebroprotective effect of dexmedetomidine by upregulating the expression of miR129, thereby attenuating hypoxic-ischemic brain injury [27]. In Mao’s study [27], with CoCl_2_ and hypoxia/reoxygenation treatments, the overexpression of MEG3 induced by I/R promotes apoptosis of TECs via regulation of the miR129 axis. Therefore, miR129 was speculated here to catalyze O_2_ production, such as from H_2_O_2_, which leads to adequate wound O_2_ content and finally negative feedback inhibition of HIF-1α expression.

To verify the protective effects of miR129 on HaCaT cells against radiation, the “gold standard” paradigm was adopted. A cell clone formation experiment showed that, after 4 Gy of irradiation, the relative clone number of the simple irradiation group (Group B) decreased by ~ 60% compared with before irradiation *(p <* 0.05). In comparison, the relative clone number of Group E only decreased by ~ 35% *(p <* 0.05, Fig. 6C). Moreover, because CeO_2_-NPs have free radical-scavenging activity and have been directly used to reduce radiation-induced dermatitis, the results showed that intraperitoneal injection of CeO_2_-NPs in athymic nude mice successfully reduces G-III dermatitis and skin pigmentation induced by X-radiation [28]. This finding suggests that the present application CeO_2_ and miR129 enhanced the anti-radiation ability of cells, which was conducive to the formation of cell clones.

### Effects of MS-CeO_2_-miR129 on the RISI mouse model

**Fig. 7:**
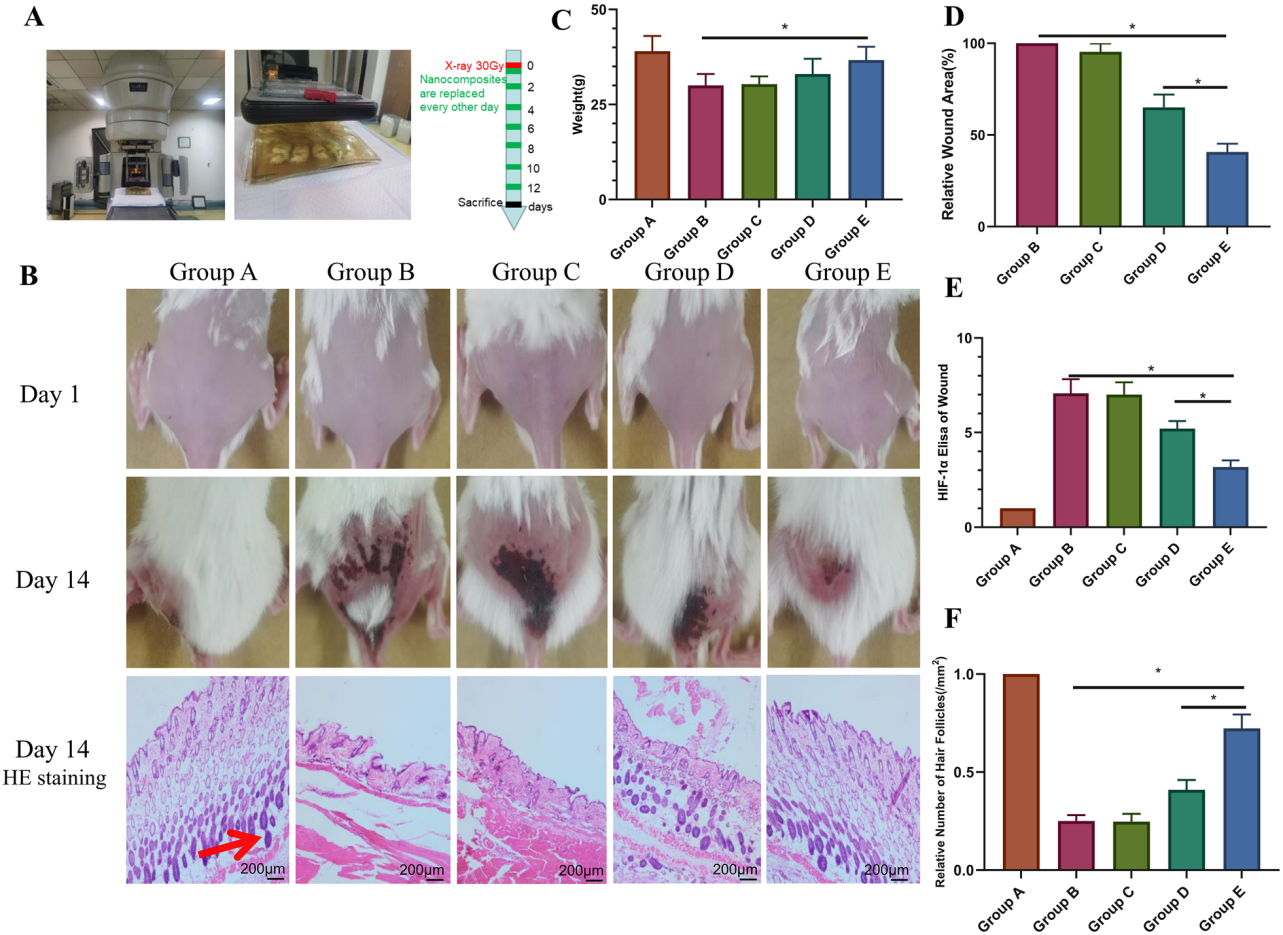
In vivo RISI mouse study of MS-CeO_2_-miR129. Medical electron linear accelerator and operation process in the mouse RISI model (**A**). Photographs of wounds on days 1 and 14 and H&E staining on day 14 (**B**). Weight (g) of mice on day 14 (**C**). Wound area for each group on day 14 (**D**). ELISA of HIF-1α for each group on day 14 (**E**). Relative number of hair follicles on day 14 (**F**). * *p* < 0.05

In the present study, the sustained efficacy of miR129 was enhanced by forming the nanocomplex MS-CeO_2_-miR129, which was delivered into the skin by subcutaneous administration before X-irradiation. This design significantly increased the stability of CeO_2_ and miR129, such that it was stabilized and activated, with CeO_2_ and miR129 subsequently released from MS-CeO_2_-miR129. The electronic beam radiotherapy equipment and the positioning and modeling process of the mouse animal model is shown in Fig. 7A. All mice were shaved and imaged to establish a model on day 1. On the 14th day, the mice were photographed again, sacrificed, and local skin tissues collected for H&E staining (Fig. 7B). Compared with Group B, Groups D and E promoted wound healing (*p* < 0.05) and the effects of Group E significantly higher than that on Group D (*p* < 0.05). The above results demonstrated that MS-CeO_2_-miR129 had the strongest effect on wound healing.

The present designed nanosystem possessed multiple advantages. (I) MS core-shell structured nanotransfersomes were suitable for drug delivery across the wound bed and the resulting entrapment of miR129 into the nanotransfersomes facilitated efficient penetration and delivery of miR129 into the stratum corneum and wound bed. This effectively ameliorated previous problems with miR129, including poor systemic absorption, low bioavailability and strong systemic clearance after oral administration. (II) Co-encapsulation of CeO_2_ into the nanotransfersomes further enhanced both wound permeation and deposition of CeO_2_-miR129. (III) Experimental results showed that MS-CeO_2_-miR129 exhibited good free radical-scavenging ability with insignificant cytotoxicity and effectively promoted cell migration, vascularization, anti-apoptosis and intracellular ROS decreases, indicating the potential application of the nanotransfersomes in protecting skin damage caused by UV radiation.

In terms of wounds on the 14th day after radiation, the wound area of mice in the MS-CeO_2_ group was ~ 65% of that in group B, while it was only 41% in the MS-CeO_2_-miR129 group, such that group E healed best, with no significant change in body weight (*p* < 0.05, Fig. 7C, D). Some gene-based drugs have been shown have potential for treating radiation-induced skin diseases, but they are unstable in vivo, which limits their application to a certain extent. Ahmed [10] has suggested that CeO_2_-NPs might be involved in cell-killing mechanisms when higher doses (50–125 µg/ml) are used. Synthesized green ceria-NPs exhibit high antioxidant activity and bactericidal effects against both G^+^ and G^−^ bacteria. In vivo studies in rats have confirmed that green ceria NPs are effective for skin wound treatment. Compared with the control group, nanoceria induce collagen deposition and increased skin tensile strength. Gobi [29] has reported that wound dressings prepared with chitosan and cellulose acetate composite and nanosized CeO_2_ show that bacteriostatic activity is controlled by varying nanoceria proportions. Films have been recommended as ideal wound dressing products. Kalantari [30] has analyzed CeO_2_-NPs developed with a green method using ginger extract to reduce the toxicity of synthesized compounds. Ceria-NPs (5 nm) have been fabricated by a freeze-thaw method in a PVA/chitosan/CeO_2_-NP hydrogel with concentrations from 0 to 1% (wt%). The antibacterial activity of the hydrogels has been investigated against wound infection bacteria, including *E. coli* and *S. aureus*. These nanocerias might be promising wound dressings because they can effectively reduce wound infection without the use of antibiotics.

HIF-1α expression in ELISA analysis showed that the expression of HIF-1α in Group E was significantly higher than that of Groups B and D (both *p <* 0.05), suggesting that the material reduced wound hypoxia by inhibiting the HIF-1α pathway (Fig. 7E). HIF-1α is a well-known central role in the hypoxic response and, under normoxic conditions, HIF-1α is susceptible to enzymatic degradation but remains highly active under hypoxic conditions. In summary, MS-CeO_2_-miR129 reduces radiation sensitivity by alleviating RISI trauma hypoxia and resisting radiation-induced DNA damage, further illustrating that MS-CeO_2_-miR129 could act as a radiation resistance agent.

During wound repair, the amount and quality of the formation of blood vessels directly affects the degree of wound healing [31]. Analysis of H&E staining results showed that the hair follicles were essentially identical (Fig. 7B, F) and, as radiation reduces hair follicle density, such decreased could lead to wounds. New epithelial growth and wound vascularization processes are inhibited, thus delaying wound healing. Here, MS-CeO_2_-miR129 significantly increased hair follicle density, promoting wound healing and improving radiation inhibition. Group E contained miR129, suggesting its potent promoting effect (*p <* 0.05). Similar to this RISI work, Zhao [8] has indicated that fullerenol, known as a “free radical sponge”, is an excellent choice for skin radiation protection because of its broad-spectrum free radical scavenging ability, good chemical stability and biological safety. In vitro results have shown that fullerenol significantly blocks ROS-induced damage and improves the viability of irradiated human keratinocytes. In vivo experiments have shown that fullererenol-loaded medical sodium hyaluronate hydrogel is suitable for dermal delivery, which can effectively protect epidermal stem cells and alleviate radiation dermatitis.

Currently, miRNAs are recognized to regulate a variety of important physiological and pathological processes, including growth, cell death and tumor formation, and to play important roles in regulating host immune responses against infections. Recently, miR129 has also been identified as a novel regulator of inflammation in different disease conditions and can be regulated through HMGB1/TLR4/NF-κB signaling, which inhibits apoptosis and inflammation [32], thereby attenuating spinal cord injury in mice. The increase in miR129 expression protected against blood reperfusion by reducing inflammation induced neuronal and blood spinal cord barrier injury via suppression of HMGB1 and TLR3-related cytokines. However, the role of miR129 in the pathogenesis of RISI is unclear. The present study showed for the first time that miR129 played a positive role in RISI wound healing.

In summary, many forms of nanomaterials have been used to treat RISI, especially for nanocarrier gene drug therapy. Meanwhile, feasible administrative pathways are also diverse. Of course, the use of subcutaneous administration, percutaneous absorption and skin penetration is first recommended in topical formulations, as the fastest ways for introducing drugs to skin tissues. In a societal sense, RISI is the most common radiation-induced disease because the skin covers the whole body and is the largest organ of all organs in the human body. External radiation beams for tumor radiotherapy first pass through the skin and then reach the tumor area. Therefore, rational design and development of nanomedicines to treat RISI are of great practical importance.

### Related mechanism of MS-CeO_2_-miR129

**Fig. 8:**
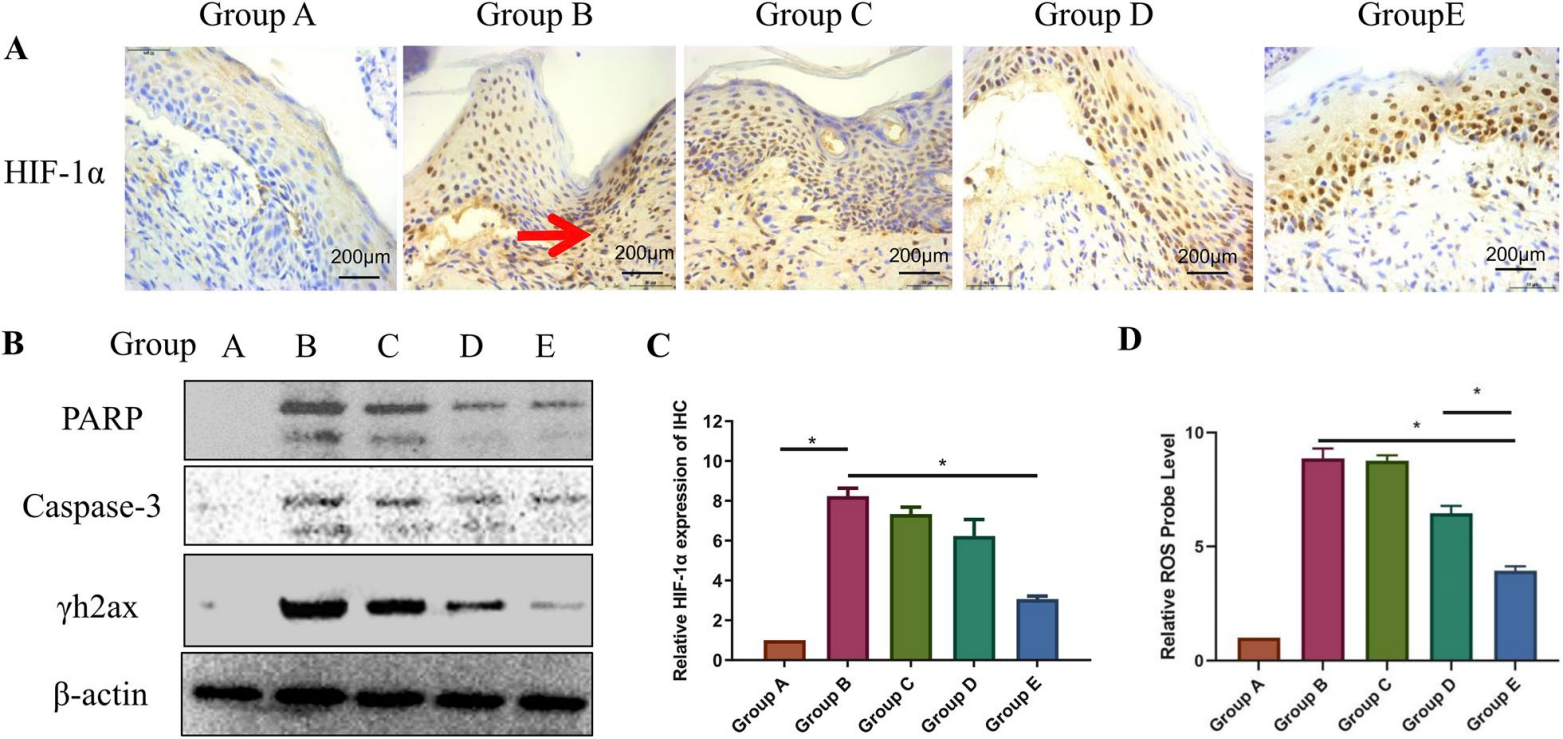
Mechanistic study of MS-CeO2-miR129. **A** Immunohistochemistry of mouse RISI on day 14. **B** Positive antibody expression (red arrow). Relative WB expression of PARP, caspase-3 and γh2ax on day 14. **C** Quantitative analysis of A. **D** Relative probe levels of ROS on day 14. *p < 0.05

A preliminary study of the material mechanism for promoting wound healing in mice was performed (Fig. 8). Many mechanistic studies have found that ROS expression is closely related to the occurrence and development of RISI [33, 34]. Zhang [35] has indicated that Cr/CeO_2_ nanozyme possesses more effective ROS scavenging and multienzyme-like properties than undoped ceria, which has been ascribed to an increased Ce^3+^/Ce^4+^ ratio. The relative probe concentrations of ROS expression increased significantly after 30 Gy of radiation, but MS-CeO_2_-miR129 (Group E) reduced ROS (Fig. 8D). ROS induced by ionizing radiation are mediators of DNA damage [33], making ROS one of the most important factors inhibiting RISI wound healing. Thus, it can prevent the wound transition from the inflammatory phase to the proliferative phase, keeping the wound in an inflammatory state. Apart from better bioavailability, the introduction of CeO_2_ provided MS-CeO_2_ with excellent antioxidant properties. It not only reduced inflammation and promoted RISI wound healing, but also exhibited great potential in applications for other chronic wounds, such as pressure ulcers, variceal ulcers of the lower extremities, and arterial occlusive acetal constriction [24].

Previous studies [36] have suggested that the benefit of radiotherapy might be due to DNA double strand breaks (DSBs), which result from energetic damage to the DNA backbone and lead to γh2ax foci formation. Zhou [37] has confirmed that overexpression of miR181a results in the accumulation of poly(ADP-ribose) polymerase (PARP), cleaved caspase-3, and increased γh2ax expression, suggesting that overexpression of miR181a might enhance radiation sensitivity by promoting DSBs. As indicated here by IHC, Group A showed less expression of HIF-1α and the DNA damage and repair-related pathway PARP/γh2ax. Also, the positive expression of related proteins (Group B) increased significantly after 30 Gy of irradiation (Fig. 8A, C). MS-CeO_2_-miR129 (Group E) had the most potent effect for promoting PARP/γh2ax and inhibition of HIF-1α expression, *(p <* 0.05). As shown in Western blots (WB) of PARP/γh2ax and caspase-3, MS-CeO_2_-miR129 also had the most potent effect in promoting PARP/γh2ax and caspase-3 expression (*p <* 0.05, Fig. 8B).

Lin [32] has indicated that the dual luciferase reporter assay indicates that Atg14 is a direct target of miR129. This illustrates that miR129 is a novel small molecule that regulates autophagy by targeting Atg14, suggesting that it might be a proinflammatory and therapeutic target for fungal keratitis. Xiong [38] has suggested that Exo from mesenchymal stem cells alleviate early brain injury after subarachnoid hemorrhage via the anti-inflammatory and anti-apoptotic effects of miR129 by suppressing the activity of the HMGB1-TLR4 pathway. Here, miR129 was found to significantly reduce DNA damage and apoptosis in RISI injury, which was demonstrated by the formation of γ-h2ax foci and elevated expression of cleaved caspase-3.These findings indicated that miR129 acts as a radiation antagonist by promoting DNA repair and inhibiting DNA damage. Consistent with IHC results, nano-miR181a also elevated cleaved PARP and γh2ax expression in WB results, indicating that MS-CeO_2_-miR129 can play a radioresistance synergistic role through DNA repair and damage.

## Conclusion

A multifunctional radioresistance strategy was developed that integrated MS-CeO_2_-based nanozymes and miR129, forming an excellent core-shell structure, with anti-ROS, anti-HIF-1α and radioresistance activity for efficient radiotherapy. MS provided the basic core-shell structure and promoted the circulation of materials in wound vessels. These MS-CeO_2_ nanozymes acted as highly efficient antimicrobial, anti-ROS and anti-hypoxia agents in RISI wounds. Importantly, the MS-CeO_2_ nanocomposite greatly reduced HIF-1α by alleviating hypoxia in vivo and in vitro, thus reducing damage by ROS. This highlights for the first time that miR129 can be shown to regulate PARP and the clinical significance of γh2ax reduction in radiosensitivity. Notably, the nanodelivery of an miR129 via MS-CeO_2_ nanocomposites was able of conferring to resistance the radiation damage in RISI models. CeO_2_ and miR129 were found to act synergistically in anti-ROS and anti-inflammation effects by activating the PARP/γh2ax signaling pathway.

MS-CeO_2_-miR129 ameliorated inflammation in RISI wounds and induced granular tissue formation, angiogenesis and collagen deposition, thus resulting in faster wound closure. In addition, MS-CeO_2_-miR129 also improved the immune microenvironment. This nanomaterial appeared able to provide a valuable alternative and promising strategy for RISI wound repair.

## Data Availability

All data generated or analyzed during this study are included in this published article.
